# Effect of forage sorghum hybrids varying in berry size on berry processing score and in situ starch digestibility

**DOI:** 10.3168/jdsc.2025-0749

**Published:** 2025-05-09

**Authors:** Douglas Duhatschek, Jourdan Bell, Luiz F. Ferraretto, Diego Duretto, John Goeser, Elizabeth Coons, Jason K. Smith, Sushil Paudyal, Juan M. Piñeiro

**Affiliations:** 1 Department of Animal Science, Texas A&M University, College Station, TX 77805; 2 Department of Soil and Crop Sciences, Texas A&M University, College Station, TX 77805; 3 Department of Animal and Dairy Sciences, University of Wisconsin, Madison, WI 53706; 4 Richardson Seeds Ltd., Vega, TX 79092; 5 Rock River Laboratory Inc., Watertown, WI 53094

## Abstract

Water scarcity threatens the production of forages used to feed lactating dairy cows. Utilizing water efficient crops is key for dairy farms trying to mitigate drought and forage scarcity risks. Forage sorghum is a water-efficient crop with the potential to partially replace corn silage. However, challenges with processing forage sorghum berries have resulted in poor starch digestibility and decreased milk production of lactating dairy cows compared with those fed with corn silage. Consequently, this has prevented its adoption by dairy farmers. The objective of this study was to evaluate if a 1.70-mm berry processing score (BPS_1.70_) and ruminal in situ starch digestibility at 7 h (isSD7) would increase when harvesting forage sorghum with larger berry size compared with regular berry sized hybrid. Two forage sorghum hybrids (F10 and F24) were seeded in 5 plots blocked by the spans across the center irrigation pivot in a randomized complete block design. The F10 hybrid had average size berries; only 41.7% of intact berries were retained above a 3.35-mm sieve. Conversely, the F24 hybrid was genetically developed to produce a larger whole berry size and retained 90% of intact berries above a 3.35-mm sieve. With larger berries, the proportion of starch for the F24 increased almost 3 percentage units compared with F10 (26.6% and 23.9%, respectively). However, BPS_1.70_ and isSD7 were not different. The study suggests that although increasing berry size may increase starch content, it does not necessarily increase BPS_1.70_ and isSD7, at least for the berry size difference tested and harvested with kernel processors and settings commonly used to process corn.

## Highlights

• Increasing berry size may increase forage sorghum starch content.
• Increasing berry size did not increase 1.70-mm berry processing score.
• Increasing berry size did not increase in situ starch digestibility at 7 hours.

## Graphical Abstract

**Figure:**
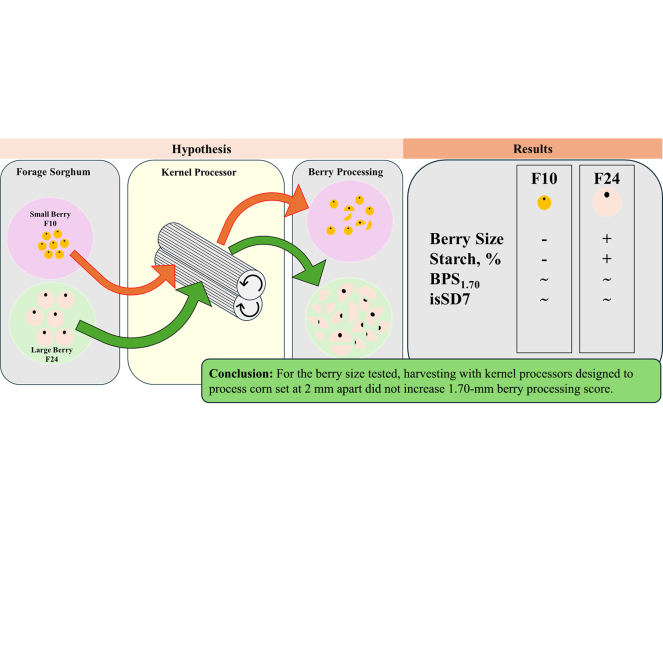
**Summary:** We evaluated the effect of forage sorghum berry size on berry processing score (BPS) at harvest, aiming to improve in situ starch digestibility. Forage sorghum with larger berry size had higher starch content; however, in situ starch digestibility after 7 hours of incubation (isSD7) was not improved. For the berry sizes tested, harvesting with kernel processors designed to process corn set at 2 mm apart did not increase the 1.70-mm BPS (BPS_1.70_) and in situ starch digestibility.

The lower starch digestibility of forage sorghum silage compared with corn silage is a major drawback preventing wider inclusion of sorghum silage in lactating dairy cow diets. Processing sorghum grain improves starch digestibility because it disrupts the pericarp and exposes the granules of starch while also increasing the surface area in which ruminal microbes and enzymes can attach to start hydrolyzation (Johnson, 2017; McCary, 2019). A linear increase in ruminal in situ starch disappearance was observed as sorghum berries were cut in smaller pieces (15.2%, 22.6%, and 39.7% of starch for whole, 2, and 4 pieces, respectively; McCary, 2019).

The small berry size of sorghum poses a greater processing challenge at harvest compared with corn. Sorghum berries are smaller than corn kernels. The geometrical mean particle size of sorghum is roughly 28% of that of corn grain (2,152 µm^2^ vs. 7,556.7 µm^2^ for sorghum and corn grain, respectively; Dias Junior et al., 2016; McCary, 2019). Dias Junior et al. (2016) reported that 100% of corn kernels were retained above the 4.75-mm sieve, indicating that the corn grain shortest dimension is greater than 4.75 mm (Dias Junior et al., 2016; McCary, 2019). Conversely, no sorghum berries were retained on the 4.75-mm sieve, and 97.5% of intact sorghum berries were retained above the 2.36-mm sieve (McCary, 2019). This would suggest that most sorghum berries, with a quasispheroidal shape, have a diameter greater than 2.36 mm.

The extent of kernel processing in corn can be assessed with the kernel processing score (**KPS**), defined as the proportion of starch passing through a 4.75-mm sieve (Ferreira and Mertens, 2005). However, because sorghum berries are smaller than corn kernels, the KPS does not properly reflect sorghum berry processing. Therefore, Johnson (2017) adapted the KPS to a berry processing score (**BPS_1.70_**), defined as the percentage of starch passing through a 1.70-mm sieve. However, McCary (2019) proposed the percentage of starch passing through a 2.36-mm sieve as a revised BPS (**BPS_2.36_**). This is because in their study, 73.9% and 14.1% of DM of berries cut into 4 pieces were retained above the 1.7-mm and 2.36-mm sieves, respectively; suggesting a potential underestimation of berries processed in 4 pieces when utilizing the 1.7-mm sieve as a threshold (McCary, 2019).

In corn harvested for silage, kernel processing can be increased through decreasing the theoretical length of cut and changing the roll gap of kernel processors (**KP**; Johnson et al., 1999; Cooke and Bernard, 2005). However, this would decrease harvest efficiency and increase fuel consumption (Johnson, 2017). This is even more challenging with forage sorghum, where the smaller berry size magnifies grain processing inefficiencies compared with corn. McCary (2019) increased BPS_1.70_ when harvesting sorghum with shorter theoretical length of cut (**TLC**) and 1-mm KP roll gap compared with a KP roll gap of 3 mm; however, the largest BPS_1.70_ achieved of 25% was still far from the optimal 50% of starch passing the 1.7-mm sieve suggested by Johnson (2017).

Increasing the berry size of sorghum for silage may increase berry processing at harvest, in turn improving starch digestibility without a need to change the equipment settings from those used for corn, and thus without impairing harvest efficiency. Although berry size is influenced by agronomic management, it is largely controlled by specific genetic traits (Baye et al., 2022; Wondimu et al., 2023). Therefore, the objectives of this study were to compare berry processing score and in situ starch digestibility of 2 forage sorghum hybrids with different berry sizes when harvested with conventional KP and harvest settings commonly used to harvest corn silage. We hypothesized that a forage sorghum hybrid harvested with a larger berry size would have greater berry processing score and increased in situ starch digestibility compared with a forage sorghum hybrid that yields conventional berry size when harvested with a TLC of 19-mm and 2-mm KP roll gap settings.

The effect of forage sorghum berry size was assessed comparing 2 hybrids (large berry size [**F24**] and regular berry size [**F10**]; Richardson Seeds, Vega, TX) in a randomized complete block design replicated 5 times. Hybrids were randomly assigned to 5 plots each after blocking. Blocks were spans across the irrigation pivot. Plots were sown in a field under central pivot irrigation at a commercial dairy farm in Amherst, Texas, on May 24, 2021. We compared a forage sorghum that produces a larger berry size (F24) to a forage sorghum that produces a regular berry size (F10). Both hybrids were conventional forage sorghum, without the brown midrib trait. The average plot size area ± SD was 1,700 ± 74.6 m^2^, with an average length of 186 ± 8.2 m, and 9.14 m in width. The seeding rate was 5.6 kg/ha.

Pre-harvest plant phenotypical characterization was performed by manually harvesting 10 plants per plot the day before harvest. Plants were cut 20 cm above the ground. The panicle was separated from the leaf and stems of each plant and dried in a forced-ventilation oven at 65°C for at least 2 d until constant weight was achieved. Plant parts were weighed separately to determine the proportion of panicle dry weight relative to plant biomass dry weight. Berries were separated from the panicles and placed on a Ro-Tap sieve shaker (W.S. Tyler, Mentor, OH) with 4 sieves (5.6-, 4.75-, 4.0-, and 3.35-mm) and a pan. The proportion of berries retained above the 4.0-mm sieve, within the 4.0- and 3.35-mm sieves, and passing the 3.35-mm sieve were weighed and used for statistical analysis.

Sorghum hybrids were monitored biweekly to assess plant maturity by visual observation the of seed coat and the ability to crush berries between the thumb and index fingers. Both hybrids were harvested on September 3 (102 d after seeding) at the soft dough stage (29.8% ± 1.6% DM; average ± SD). The self-propelled forage harvester (9800i, John Deere, Moline, IL) had KP designed to process corn (XStream, John Deere, Moline, IL) set 2 mm apart with 50% differential speed and a TLC of 19 mm. The KP had harvested ∼20,000 Mg before the experiment, and the aperture gap was measured across the entire length of the rolls. At harvest, duplicate fresh samples were taken from each plot after the truck unloaded the forage by the silage pile. Samples were immediately placed in a cooler with ice until being frozen. Forage yield per plot was calculated from the entire plot area by subtracting the empty truck weight from the total weight before unloading the forage. Forage yield was then corrected for DM to have an estimated forage DM yield.

Frozen subsamples were sent to Rock River Laboratory (Watertown, WI) for nutrient composition analyses and berry processing score evaluations, and to University of Wisconsin–Madison (Madison, WI) for in situ starch digestibility assays at 7 h. Dry matter was assessed by drying the samples in a forced-ventilation oven. Crude protein, ADF, lignin, and ash were estimated with near infrared spectroscopy (**NIRS**) analysis. Amylase-treated NDF (**aNDF**) and NDF digestibility at 30 h (**NDFD30**) were assayed through wet chemistry with the Ankom method for aNDF and in vitro incubations procedures described by Goeser et al. (2009) for NDFD30. Samples were dried as described previously and ground to 1-mm (Cyclone sample mill; UDY Corporation, Fort Collins, CO) before being analyzed by NIRS using a Foss 5000 near-infrared spectrometer (Foss North America Inc., Eden Prairie, MN). Calibration equations for NIRS analysis of CP, starch, ADF, lignin, and ash were based on the following wet chemistry procedures: Dumas method for CP (modified AOAC 967.05; AOAC International, 2016); Enzymatic reaction for starch (modified AOAC 2014.10); Ankom method for ADF; the use of sulfuric acid to isolate lignin from ADF (modified AOAC 973.18); and combustion method at 550°C during 4.5 h for ash (modified AOAC 942.05). Berry processing score was evaluated as the proportion of starch passing the 1.70-mm (BPS_1.70_) or 2.36-mm sieve (BPS_2.36_; Johnson, 2017; McCary, 2019).

The in situ incubations were conducted under an approved protocol by the Animal Care and Use Committee of the College of Agriculture and Life Sciences at the University of Wisconsin–Madison. Ruminal in situ incubations were performed using 2 mid-lactation Holstein cows (167 and 232 DIM), which were eating a diet with 26% starch. Undried and unground samples were used to maintain the effects of berry processing on ruminal in situ starch disappearance. Approximately 5.00 ± 0.28 g of DM of each sample were placed into polyester bags (10 × 20 cm, 50 ± 10 μm porosity; R1020, Ankom Technologies). The bags were placed in laundry bags and incubated in the ventral sac of the rumen. Each sample was incubated for 7 h in duplicate within each cow. After removal, sample bags were submerged in cold water (water with ice) for 15 min and rinsed with room temperature tap water to wash off any large particles adhered to the bags. Sample bags were then placed in clean laundry bags for further washing in a washing machine set on the rinse and spin cycle with room temperature water for 30 min. Dry residue from each sample within cow was combined and ground to pass a 1-mm sieve in a cyclone mill. The ground residue was then analyzed for starch concentration by an enzymatic method (Hall, 2015) with thermostable α-amylase (Ankom Technology, Macedon, NY) and amyloglucosidase (Megazyme E-AMGDF, Bray, Co. Wicklow, Ireland) enzymes.

Generalized mixed models were performed using the MIXED procedure of SAS (SAS version 9.4; SAS Institute Inc.) to evaluate the effect of forage sorghum hybrids with different mean berry sizes on berry processing score (1.70- and 2.36-mm sieve) and ruminal in situ starch digestibility at 7 h. Block was included as the random variable in all models. Residuals were assessed with the UNIVARIATE procedure of SAS 9.4 and considered normally distributed (Shapiro–Wilk test, W ≥ 0.85). A *P*-value ≤0.05 was considered statistically significant.

Forage sorghum plant composition, berry size, grain yield, and total yield are summarized in Table 1. Berry size was greater for the hybrid F24 compared with F10. The F24 retained more pre-harvest grain above the 4.0-mm sieve (0% vs. 41% for F10 and F24, respectively; *P* = 0.008) and less grain below the 3.35-mm sieve compared with F10 (58% vs. 10% for F10 and F24, respectively; *P* = 0.02). However, the proportion of panicle dry weight was greater for F10 than F24 (*P* = 0.03).

**Table 1:** Pre-harvest phenotypical plant parts evaluation, and harvest DM yield of forage sorghum hybrids (F10 and F24)

Item	F10	F24	SEM	*P*-value
Intact berries particle size distribution, %				
>4.0 mm	0.0	41.0	2.59	0.008
4.0–3.35 mm	41.5	48.7	3.96	0.24
<3.35 mm	58.5	10.3	4.51	0.02
Yield, Mg of DM/ha	13.2	13.9	0.47	0.33
Plant dry weight, g	114.2	155.1	8.84	0.01
Dry weight panicle proportion, %	48.8	44.4	1.16	0.03

Table 2 summarizes the nutrient composition analysis, and NDFD30 of both hybrids. Dry matter, aNDF, ADF, and ash were not different between treatments. Lignin was lower for F24 than F10 (4.08% ± 0.08% and 4.44% ± 0.08%, respectively; *P* = 0.0003). However, NDFD30 did not differ (*P* = 0.17). Crude protein content was greater for F10 than F24 (9.95% and 8.94% ± 0.09%, respectively; *P* < 0.0001). In addition, F24 had greater concentration of starch when compared with F10 (26.6% ± 1.02% of DM and 23.9% ± 1.02% of DM, respectively; *P* = 0.01).

**Table 2:** Nutrient composition of forage sorghum with regular berry size (F10) and larger berry size (F24)

Item	F10	F24	SEM	*P*-value
Dry matter, %	29.7	29.8	0.67	0.88
Crude protein, % of DM	9.95	8.94	0.09	<0.0001
Starch, % of DM	23.9	26.6	1.02	0.01
aNDF, % of DM	44.2	44.0	0.61	0.77
ADF, % of DM	29.8	29.2	0.53	0.22
Lignin, % of DM	4.44	4.08	0.01	<0.001
Ash, % of DM	8.56	8.58	0.13	0.91
NDFD30, % of NDF	44.5	45.5	0.53	0.17

Berry processing score and in situ starch digestibility are displayed in Table 3. Although the berry size and starch content were greater for F24 than for F10, BPS_1.70_ was not different. However, the amount of starch passing the 2.36-mm sieve was greater for F10 compared with F24 (32.1% ± 1.4% and 24.6% ± 1.4%, respectively; *P* = 0.0004). Nonetheless, in situ starch digestibility was similar between treatments.

**Table 3:** Berry processing score and in situ starch digestibility of forage sorghum with regular (F10) and large (F24) berry size after harvesting

Item	F10	F24	SEM	*P*-value
Starch passing sieve, %				
Sieve, mm				
<1.7^1^	14.9	15.8	0.79	0.34
<2.36^2^	32.1	24.6	1.40	<0.001
In situ starch digestibility, % of starch	59.5	59.3	3.49	0.97

1 BPS_1.70_.

2 BPS_2.36_.

The increased berry size of sorghum hybrid F24 was demonstrated with proportionally more pre-harvest whole berries retained above the 4-mm sieve compared with F10 (41% versus 0%, respectively; *P* = 0.008; Table 1). In addition, F10 had more intact berries passing the 3.35-mm sieve before harvesting (58.33% and 10.33% for F10 and F24, respectively; *P* = 0.02). McCary (2019) reported 80.4% of whole berries passing through the 3.35-mm sieve using 25 hybrids at different maturities, suggesting that F24 also may have had larger berry size than average sorghum hybrids.

Hybrid F24 had a higher plant dry weight than F10, but this did not translate into a significantly higher biomass yield. This may be due to lower germination and plant density per unit of land for F24 compared with F10. However, a limitation of this study is that plant stand counts were not assessed.

Although F24 had larger berries, it was ∼0.4 m taller and had proportionally greater dry stover weight but lower dry panicle weight than F10. This suggests that factors other than grain yield influenced the higher starch content in F24 compared with F10. The F24 larger grain size would result in a greater volume-to-surface ratio and proportionally more endosperm, where starch is the major component, and less pericarp that contains mainly ADF and aNDF (Santiago-Ramos et al., 2018). Therefore, the increased starch content in F24 compared with F10 (*P* = 0.01) may be partially explained by the larger grain size and not by differences in developmental stages of grain production because both hybrids were harvested at the same physiological stage, and with similar DM.

Despite the increased berry size, the grain size of F24 was not large enough to improve BPS_1.70_ with the conventional KP and harvesting settings typically used for corn silage harvesting compared with F10. Therefore, in situ starch digestibility was also not different between hybrids. Berry processing score 1.7 mm was 14.9% and 15.8% ± 0.79% for F10 and F24, respectively (*P* = 0.34). Johnson (2017) conducted 2 studies using silage samples collected from commercial dairy farms in Kansas. He reported a linear increase in BPS_1.70_ as the KP roll gap aperture decreased. In study 1, BPS_1.70_ increased from 18.9% to 36.9% (±2.9%) of starch, and in study 2, from 26.3% to 55.1% (±4.0%) of starch. In the present experiment, KP was set at 2-mm roll gap, which is wider than roll gap apertures in other studies with sorghum silage and resulted in a similar BPS_1.70_ to the unprocessed sorghum silage tested by Johnson (2017). Future studies should assess the effect of increasing berry size on BPS_1.70_ using a narrower KP gap setting.

Another experiment evaluating the effect of theoretical length of cut (16 and 22 mm) and KP roll gap (1 and 3 mm) reported an interaction between TLC and KP roll gap, where 16-mm TLC with a 1-mm roll gap increased BPS_1.70_ (25.6% of starch) when compared with 16- and 22-mm TLC with a 3-mm KP roll gap (McCary, 2019). Despite the TLC and KP roll gap in the present study being between the roll gap and TLC treatments applied by McCary (2019), BPS_1.70_ was lower in the current experiment compared with all treatment groups in their study. This could be due to different design and wear of KP, or differences in hybrids, plant maturity stages, and humidity at harvest. An interaction between maturity at harvest and the use of KP was reported when harvesting corn silage, where the late maturity corn (DM = 49.2%) harvested with KP had the largest KPS when compared with late maturity without KP and earlier maturity corn (DM = 43.1%) harvested with and without KP (Hamilton et al., 2021). Similarly, an increase in KPS was reported for corn silage harvested at later maturity with a 1-mm KP roll gap compared with corn silage harvested at earlier maturity with the same harvest settings (Saylor et al., 2021). It is possible that the lower BPS_1.70_ in the present study compared with the one reported by McCary (2019) was due to maturity indicated by the lower DM content in the present experiment (<30% vs. >36% DM). We speculate that a drier grain would be more likely to break when passing through KP with a narrow roll gap because of the reduced malleability of the grain compared with a wetter grain in an earlier plant maturity stage, which could squeeze by when passing through the rolls and thus reduce BPS_1.70_.

The BPS_2.36_ was greater for F10 than F24 even though BPS_1.70_ did not differ. Increased BPS_2.36_ observed for the smaller F10 berries compared with F24 may be consequence of a larger proportion of F10 intact or halved berries passing though the 2.36-mm sieve, rather than better processing. Similarly, a study found that 2.54%, 51.4%, and 85.9% of whole, halved, or quartered berries passed through the 2.36-mm aperture sieve, respectively (McCary, 2019). Conversely, in our study, the larger F24 berries would have to be processed into more pieces to pass through the same sieve aperture compared with the smaller F10 sorghum berries.

Regardless of the difference observed in BPS_2.36_ between the hybrids tested, BPS_2.36_ was poor and BPS_1.70_ and isSD7 did not differ. We speculate this could be due to a similar proportion of F10 and F24 intact berries. However, one limitation of this study is that we did not assess the proportion of intact berries as another measurement of berry processing. The pericarp is a physical barrier that impedes ruminal microbes access from accessing granules of starch, reducing starch digestibility (McAllister et al., 1994). Thus, berry processing is essential to disrupt the pericarp and expose the starch for microbial and enzymatic digestion. Optimal BPS_1.70_ for sorghum was suggested to be above 50%, which is much greater than what was obtained in this experiment (Johnson, 2017).

Forage sorghum with larger berry size (F24) had a greater starch concentration than a regular berry size sorghum hybrid (F10) harvested at the same physiological stage. Although forage sorghum hybrid F24 produced larger berries, we found no difference in BPS_1.70_ and in situ starch digestibility at 7 h incubation of freshly chopped material when compared with the regular berry size forage sorghum hybrid (F10). However, the current study used KP and settings designed to process corn. Future studies should assess the effect of increasing sorghum berry size but with KP and settings designed to maximize sorghum berry processing.

Data indicate that growing forage sorghum hybrids that produce larger berries for silage may increase the starch content of the silage but may not necessarily increase BPS_1.70_ and in situ starch digestibility when harvested with KP developed for corn silage processing and with a roll gap setting of 2 mm.

## References

[bib1] AOAC International (2016).

[bib2] Baye W., Xie W., Xie P. (2022). Genetic architecture of grain yield-related traits in sorghum and maize. Int. J. Mol. Sci..

[bib3] Cooke K.M., Bernard J.K. (2005). Effect of length of cut and kernel processing on use of corn silage by lactating dairy cows. J. Dairy Sci..

[bib4] Dias G.S., Ferraretto L.F., Salvati G.G.S., de Resende L.C., Hoffman P.C., Pereira M.N., Shaver R.D. (2016). Relationship between processing score and kernel-fraction particle size in whole-plant corn silage. J. Dairy Sci..

[bib5] Ferreira G., Mertens D.R. (2005). Chemical and physical characteristics of corn silage and their effects on in vitro disappearance. J. Dairy Sci..

[bib6] Goeser J.P., Hoffman P.C., Combs D.K. (2009). Modification of a rumen fluid priming technique for measuring in vitro neutral detergent fiber digestibility. J. Dairy Sci..

[bib7] Hall M.B. (2015). Determination of dietary starch in animal feeds and pet food by an enzymatic-colorimetric method: Collaborative study. J. AOAC Int..

[bib8] Hamilton T., Walker J., Rusche W.C., Smith Z.K. (2021). Effects of harvest maturity and/or kernel processing on corn silage processing score and particle size of corn silage. J. Anim. Sci..

[bib9] Johnson J.R. (2017).

[bib10] Johnson L., Harrison J.H., Hunt C., Shinners K., Doggett C.G., Sapienza D. (1999). Nutritive value of corn silage affected by maturity and mechanical processing: A contemporary review. J. Dairy Sci..

[bib11] McAllister T.A., Bae H.D., Jones G.A., Cheng K.J. (1994). Microbial attachment and feed digestion in the rumen. J. Anim. Sci..

[bib12] McCary C.L. (2019).

[bib13] Santiago-Ramos D., Figueroa-Cárdenas J.D., Mariscal-Moreno R.M., Escalante-Aburto A., Ponce-García N., Véles-Medina J.J. (2018). Physical and chemical changes undergone by pericarp and endosperm during corn nixtamalization—A review. J. Cereal Sci..

[bib14] Saylor B.A., Diepersloot E.C., Heinzen C., McCary C.L., Ferraretto L.F. (2021). Effect of kernel breakage on the fermentation profile, nitrogen fractions, and in vitro starch digestibility of whole-plant corn silage and ensiled corn grain. JDS Commun..

[bib15] Wondimu Z., Dong H., Paterson A.H., Worku W., Bantte K. (2023). Genome-wide association study reveals genomic loci influencing agronomic traits in Ethiopian sorghum (*Sorghum bicolor* (L.) Moench) landraces. Mol. Breed..

